# Repeat Sampling of Female Passerines During Reproduction Reveals Surprising Higher Plasma Oxidative Damage During Resting Compared to Active State

**DOI:** 10.1093/icb/icad120

**Published:** 2023-09-12

**Authors:** Kyle Coughlan, Edyta T Sadowska, Ulf Bauchinger

**Affiliations:** Institute of Environmental Sciences, Faculty of Biology, Jagiellonian University, Gronostajowa 7, 30-387 Kraków, Poland; Institute of Environmental Sciences, Faculty of Biology, Jagiellonian University, Gronostajowa 7, 30-387 Kraków, Poland; Institute of Environmental Sciences, Faculty of Biology, Jagiellonian University, Gronostajowa 7, 30-387 Kraków, Poland; Nencki Institute of Experimental Biology, Polish Academy of Sciences, 3 Pasteura St., 02-093 Warsaw, Poland

## Abstract

Traditional models of oxidative stress predict accumulation of damage caused by reactive oxygen species (ROS) production as highly correlated with aerobic metabolism, a prediction under increasing scrutiny. Here, we repeat sampled female great tits (*Parus major*) at two opposite levels of energy use during the period of maximum food provisioning to nestlings, once at rest and once during activity. Our results were in contrast to the above prediction, namely significantly higher levels of oxidative damage during rest opposed to active phase. This discrepancy could not be explained neither using levels of “first line” antioxidant enzymes activity measured from erythrocytes, nor from total nonenzymatic antioxidant capacity measured from plasma, as no differences were found between states. Significantly higher levels of uric acid, a potent antioxidant, were seen in the plasma during the active phase than in rest phase, which may explain the lower levels of oxidative damage despite high levels of physical activity. Our results challenge the hypothesis that oxidative stress is elevated during times with high energy use and call for more profound understanding of potential drivers of the modulation of oxidative stress such as metabolic state of the animal, and thus also the time of sampling in general.

## Introduction

Oxidative status is essentially the relationship between oxidants and antioxidant capacity within an organism. An imbalance between oxidants and antioxidants in favor of the oxidants, potentially leading to damage, has been defined as oxidative stress (Sies 1997). One of the most important and studied oxidant types are the reactive oxygen species (ROS), a naturally occurring and unavoidable by-product of aerobic metabolism (Murphy 2009). Oxidative stress has long been considered to play an important role in many factors affecting life-history of animals (Monaghan et al. 2009; Costantini 2014), ranging from tissue damage accumulation (Gerschman et al. 1954) and ageing (Harraan 1956), to fecundity and survival (Bize et al. 2008). Owing to its importance as a mediator of organism performance and fitness, we aimed to investigate the oxidative status of birds between two metabolic extremes.

### ROS production and accumulation of damage

The mitochondrial electron transport chain (ETC) is the main pathway for the creation of adenosine triphosphate (ATP) energy molecules in higher vertebrates. This process, however, is not perfect and there is a “leak” of electrons as it occurs. The majority of these leaks occurs as electrons move through complexes I and III (Barja 1999; Cardoso et al 2012), but some do originate from other sources within the ETC (Jastroch et al. 2010). These electrons react with molecular oxygen forming ROS molecules, such as the superoxide radical and hydrogen peroxide, which, left unquenched can induce damage to lipids, proteins, and DNA (Hulbert et al. 2007). It is estimated that between 1 and 6% of oxygen consumed is not reduced to water but rather reacts prematurely with the leaked electrons from the ETC to create free radicals (Ji 2008). Around 10% of ROS are created within animals’ cells in a controlled and compartmentalized manner, usually for functions such as cell signaling (Dröge 2002), while the remaining 90% are generated as a by-product of metabolic processes (Balaban et al. 2005). To this end, ROS production is a meaningful by-product of energy metabolism, which is both beneficial and damaging to the organism.

The relationships between oxygen consumption, ATP production, ROS generation, and thus risks of encountering oxidative damage, while inextricably linked, are not fully understood. It is clear than increased energy requirements due to locomotion require increased oxygen consumption; however, this is not a true measure of ATP production as the amount of ATP generated per unit oxygen consumed can vary significantly (Salin et al. 2015). In turn, the rate of ROS production in relation to oxygen consumption and ATP generation is also not clear. Oxygen consumption can increase severalfold due to increased energy requirements, but mitochondrial oxygen radical production does not increase proportionally (Barja 1999). This is in part to the transition of the mitochondria from State IV (rest state) with lower adenosine diphosphate (ADP, the precursor to ATP) and high superoxide and hydrogen peroxide production, to State III (active state) with higher ADP but lower ROS production in comparison (Navarro et al 2004). Phenotypic differences in mitochondrial aerobic metabolism may have important consequences for organismal performance and fitness traits. Typically, measurements of mitochondrial metabolism were low-throughput and cumbersome (Koch et al. 2021). In recent years, advances have allowed for a more minute, noninvasive examination of mitochondria function and the relationship between energy production and oxidative stress. For example, Stier et al. (2017) developed a method to measure mitochondrial functioning using intact erythrocytes of king penguins (*Aptenodytes patagonicus*), while further adaptation of this protocol in pied flycatchers (*Ficedula hypoleuca*) has demonstrated both the repeatable of this method, as well as the rapid within-individual adjustments of these traits during changing life-history stages (Stier et al. 2019). Studies such as these open up interesting avenues of research for ecologists and evolutionary biologists from variation in the subcellular energy flow to the whole-organismal level traits, including oxidative stress (reviewed in Koch et al. 2021).

Traditionally, the “oxidative stress-life history theory” as adopted by ecologists conceptualizes free radical production in direct proportion to metabolic rate, often an oversimplification (Speakman and Garratt 2014 provides a discussion of the misgiving and misuse of this theory). Central to this model is the idea of trade-offs and their presumed physiological costs (Zera and Harshman 2003). In short, animals use oxygen to release energy and thus must deal with ROS production, so increasing this metabolism, that is, during reproduction, leads to an inescapable increase in free-radical production. So, while the hypothesis that physical activity affects oxidative balance is strongly supported (Powers and Jackson 2008), the relationship between accumulation of ROS damage in line with energy use and ROS production is poorly understood (Monaghan et al. 2009).

### Defence against oxidative damage

Animals have several mechanisms for the prevention of accumulation of ROS damage, known cumulatively as the antioxidant system, reviewed in Monaghan et al. 2009. These are (1) reducing uncontrolled ROS generation and release via mediation of mitochondrial redox states (described above) or uncoupling of oxygen consumption and ATP production. (2) Three “first line” antioxidant enzymes present within the mitochondria and cells attempt to counteract the effects of superoxide anions and its derivatives before they can cause damage to molecules and lead to an oxidative cascade. These enzymes are superoxide dismutase (SOD), glutathione peroxidase (GPx; which acts in tandem with glutathione), and catalase (CAT) (see also Skrip and McWilliams 2016 and Ighodaro and Akinloye 2018 for further details). (3) Use of circulating antioxidants such as vitamin C or E, carotenoids, and uric acid to break the oxidative cascade, which occurs as ROS cause damage and create additional reactive molecules. In response to increases in ROS, organisms can upregulate endogenous production of these antioxidants and stored dietary antioxidants can be mobilized (Surai 2002). Also considered part of the antioxidative defense systems described by Monaghan et al. 2009 are (4) differing structural make up of cellular macro-molecules and (5) DNA, protein, and lipid repair or replacement mechanisms.

In the last decade, focus has moved toward more integrative measurements of the whole organismal redox systems. Pairing nonenzymatic antioxidant capacity with enzymatic antioxidant activity is emerging as an important research strategy owing to the fact that both portions of the antioxidant system appear to work in concert through different mechanisms (Costantini et al. 2011a; Skrip and McWilliams 2016, McWilliams et al. 2021).

### Background for study aims, hypotheses, and predictions

Many studies have aimed to uncover the links of energy use, ROS production, and damage accumulation through various methods such as climatic alterations, limitation of resources, workload manipulations, or comparisons of breeding and nonbreeding animals. In relation to effects of energy use and oxidative damage, in zebra finches (*Taeniopygia guttata*), it was found that birds in a high flight activity group had higher levels of damage than those in a control flight group (Costantini et al. 2013), while an experimental manipulation using simulated territorial intrusions in rufous horneros (*Furnarius rufus*) found no difference in oxidative damage between those performing 20 min of territorial defense and non-challenged birds (Mentesana and Adreani 2021). In both these studies, antioxidant defense levels decreased with increased activity; however, feather clipped great tits (*Parus major*) showed a moderate increase in oxidative damage while strongly increasing antioxidant capacity (Vaugoyeau et al. 2015). Similarly, conflicting results are seen in mammals. For example, mice (*Mus musculus*) raising offspring showed an increase in damage compared to non-reproducing females; however, in reproducing females, oxidative damage decreased with litter size at birth but increased with litter size at weaning (Stier et al. 2012), while non-bred Wistar rats (*Rattus norvegicus*) exhibited higher oxidative damage in the kidneys at 6 months old than those which had bred, but the opposite effect was found when measured at 3 and 12 months old (da Silva et al. 2013). In short, different studies have used different markers of oxidative stress, antioxidant capacity, different tissue types, and treatments and the resulting conclusions have often varied wildly (see Stier et al. 2012, Speakman and Garratt 2014; Blount et al. 2016, and Cooper-Mullin and McWilliam 2016 for reviews).

For the present study, we repeat sampled female great tits during the period of peak food provisioning in two opposite and “extreme” states of energetic metabolic “states.” Blood samples were taken before sunrise while birds were at rest during the night (Rest phase) and during the day while birds were actively providing food for their nestlings (Active phase). To the best of our knowledge, repeat sampling for oxidative stress parameters using the extremes of metabolic activity has not been performed before on wild vertebrates. Given the current understanding and knowledge on energy use and oxidative stress, there were two opposite hypotheses and accompanying predictions, which we aimed to test with this study; (1) *Energy metabolism during active daytime food provisioning is substantially higher than during rest, as such ROS production will also be higher*. Based on this, we predict that *Levels of oxidative damage will be higher when birds are sampled during activity as opposed to rest*. In contrast to the above, we also wished to examine the hypothesis that (2) *Birds have evolved antioxidant counter measures to compensate for increased ROS production during periods of heightened energy use* and as such predict that *antioxidant defense mechanisms, be they enzymatic or nonenzymatic, will be higher during activity than during rest and thus oxidative damage will either be lower or at least not different during activity than during rest*.

Although differences in energy metabolism are likely to be influenced by circadian patterns, we believe by sampling at these two time points allowed us to gain a representative measure of oxidative status of the birds at the highest and lowest points of energy metabolism experienced by the birds during their daily cycle.

## Materials and methods

### Study area and sample collection

The study was carried out in the summer of 2021, in a mixed forest near the town of Mikołajki situated in the Masurian Lake District in Northern Poland (53°47′09.86″N 21°34′53.26″E). Nest boxes (*n* = 500) were monitored throughout the 2021 breeding season. This study relates to adult birds during their confirmed second round of reproduction, which were sampled at two different levels of activity, that is, at *Rest* and while *Active* during food provisioning. Herein, we refer to Rest as samples taken at the end of the night and Active to those taken during daily food provision.

Upon hatching, nests were randomly assigned to sampling states on Day 10 and 12, with the first and second samples alternating between these activity levels. Feeding activity is known to peak around Day 8 or 9 and plateau until fledging (Drent and Daan 1980). Nests were approached half an hour before civil twilight to grab the female from nest by hand at rest, that is, with females in their assumed resting metabolism. Blood was collected immediately under red light at real time 03:06 AM ± 12 min, which corresponds to a sampling time of 25 ± 19 min before civil sunrise. For capturing the birds while active a “foil trap” was placed inside the nest and parents caught when providing food to their offspring. Average real time sample was 10:17 AM ± 18 min, which corresponds to a sampling time of civil sunrise + 6 h ± 23 min. Once the traps were in place, the observers left the vicinity of the nest box and an observer returned every 15 to 20 min to check if the birds had entered the nest and the trap was left in place a maximum of 1 h. Handling time for active samples, measured as time from trap placing until sampling, was 30 ± 24 min. Time from bird in hand until sample collection was roughly 3 min for both active and rest samples. Samples were collected following measurements of the bird by piercing the brachial vein and collecting blood in heparinized 75 µl heparinized capillary tubes. Two tubes were collected, and blood was transferred to a heparinized 0.5 ml Eppendorf tube that was stored on ice until for transport back to the lab where the samples were centrifuged (*g* × 6000 for 10 mins) to separate erythrocytes from plasma. The erythrocytes and plasma samples were stored on dry ice in a polystyrene box inside a freezer until the end of the field season when they were transported on dry ice to the Jagellonian University in Krakow and stored at −80°C until analysis. In total, 12 females were sampled during both activity phases. Further information in relation to sampling area and procedures are provided in the Supplementary Material.

### Oxidative stress measurements and laboratory analyses

Plasma was analyzed for nonenzymatic antioxidant capacity, using the OXY kit, and for oxidative damage, measured as reactive oxygen metabolites (ROMs) using the d-ROMs kit. In addition, uric acid levels were measured using a standard kit. Owing to the presence of functional mitochondria within avian erythrocytes, erythrocyte lysate was used to measure the activity or levels of three antioxidant enzymes (Superoxide dismutase: SOD, Glutathione peroxidase: GPx, and Catalase: CAT) along with the small weight antioxidant molecule glutathione. Total glutathione (tGSH) and its oxidized form, glutathione disulphide (GSSG) were measured directly, while the active form reduced glutathione (GSH) was calculated as tGSH minus GSSG.

All three plasma measurements were performed using commercial kits from Diacron International (Grosseto Italy) while the five erythrocyte measurements were performed using kits from Cayman Chemicals (Ann Arbour, MI). Sampled were run in duplicates and followed the manufacturers’ instructs with slight modifications to some protocols (see Supplementary Material for an exact breakdown of protocols and co-efficient of variations for each assay).

### Statistical analyses

Statistical analyses were performed using RStudio ver. 3.6.2 (R Core Team 2022). A mixed model approach was taken using the lme4/lmerTest packages in RStudio (Bates et al. 2015). All 10 measures of oxidative status were used as individual response variables with State (i.e., Rest or Active) used as the explanatory variable. Sampling date and order of sample were included as covariates in repeated measures along with ring number as a random factor. Body mass at time of sample was initially included in all models but was found to be insignificant and removed. Model assumptions were checked using the performance analytic package (Peterson et al 2018), all assumptions were met, and no data were transformed. Rest phase and active phase values were analyzed separately to check for several other effects such as handling time, sex, and time of sample (Please see Supplementary Material for details). Statistical significance was inferred using a frequentist approach where *P* < 0.05 was deemed statistically significant. Oxidative stress index was calculated as d-ROMs/OXY × 1000, according to Costantini et al. 2008. The GSH:GSSG ratio calculated according to Owen and Butterfield 2010.

## Results

### Erythrocyte antioxidant enzymes and glutathione levels

**Fig. 1.**
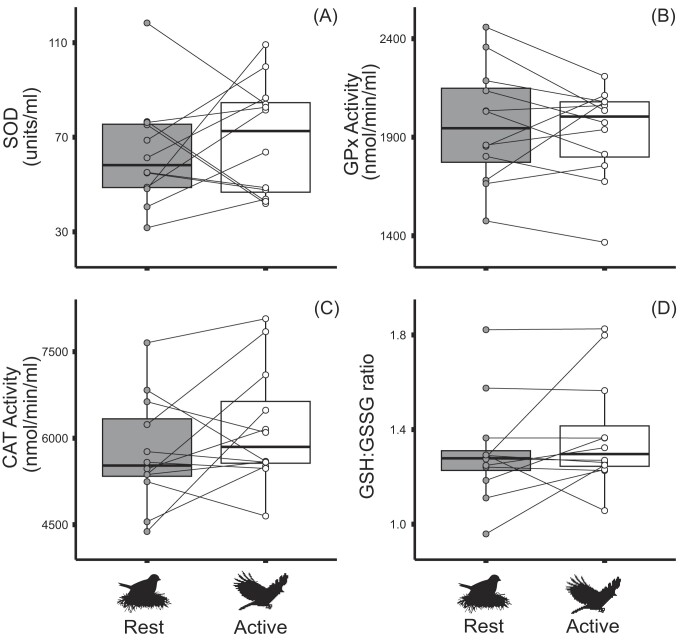
Antioxidant activity of erythrocyte lysate. The three “first line” defense antioxidants (**A**) SOD, (**B**) GPx, and (**C**) CAT (catalase) showed no significant differences between the active and rest phases. Glutathione in its reduced (GSH) and oxidized form (GSSG) also did not exhibit any differences between rest and active phases and is exhibited in the ratios between GSH and GSSG (**D**). Black line in box is median value, top and bottom hinges represent the first and third quartiles, respectively, while whiskers represent values no more or less than 1.5 times the interquartile range top and bottom, respectively. Points not falling along a whisker represent outliers.

, There was no evidence that state had any effect on antioxidant activity levels; SOD; (*F*_1,20_ = 0.46, *P* = 0.506) (Fig. 1A), GPx: (*F*_1,10_ = 0.42, *P* = 0.531) (Fig. 1B), and CAT; (*F*_1,10_ = 1.48, *P* = 0.252) (Fig. 1C). There was also no evidence that GSH or GSSG levels differed due to state, GSH; (*F*_1,10_ = 2.40, *P* = 0.152) and GSSG; (*F*_1,10_ = 0.03, *P* = 0.871), and neither was there evidence that the GSH:GSSG ratio differedt (*F*_1,10_ = 3.43, *P* = 0.094) (Fig. 1D).

### Oxidative damage

**Fig. 2.**
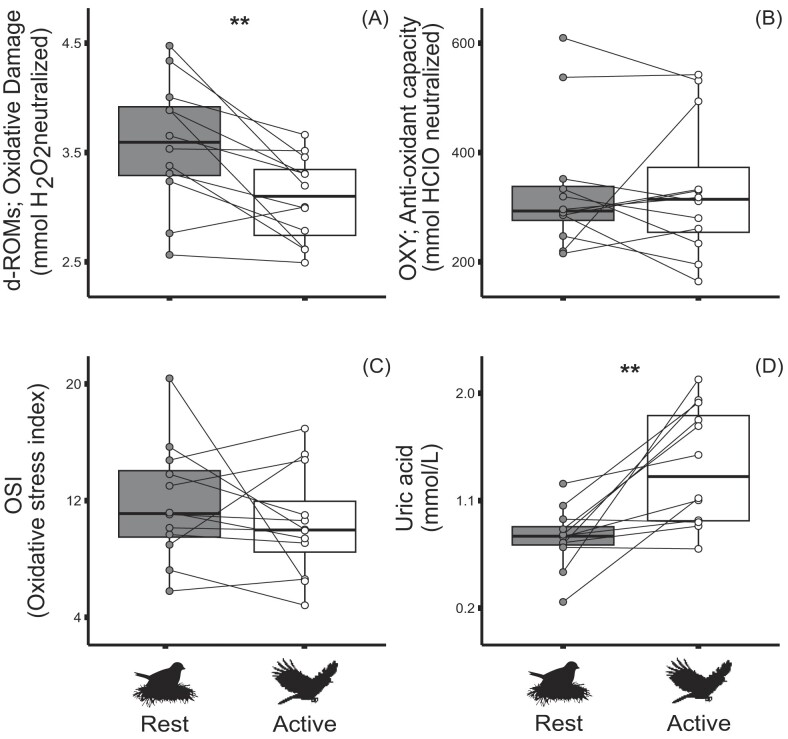
Plasma oxidative stress markers. (**A**) d-ROMs, our measure of oxidative damage was significantly higher during the night rest phase than during the daytime activity, with all but one sample higher during the night (*P* = 0.006); (**B**) OXY, nonenzymatic antioxidant capacity of plasma, showed no significant effect of activity state, as did (**C**) OSI, oxidative stress index (d-ROMs/OXY*1000). (**D**) Uric acid, a potent antioxidant present in plasma, showed a significant effect of state, being far higher during daytime activity than during the rest phase (*P* = 0.005). Black line in box is median value, top and bottom hinges represent the first and third quartiles, respectively, while whiskers represent values no more or less than 1.5 times the interquartile range top and bottom, respectively. Points not falling along a whisker represent outliers.

Contrary to our expectations, we found strong evidence that levels of oxidative damage measured using the d-ROMs test in birds were lower during their active food provisioning phase (M = 3.08, SD = 0.4) than when they were sampled out of rest (M = 3.59, SD = 0.6) (*F*_1,10_ = 12.22, *P* = 0.006) (Fig. 2A).

### Plasma nonenzymatic antioxidant capacity

There was no evidence that total circulating nonenzymatic antioxidant capacity as measured by the OXY test did not show a significant differed between states (active M = 333, SD = 126; rest M = 333, SD = 121) (*F*_1,10_ = 0.11, *P* = 0.752) (Fig. 2B). The OSI also did not differ between states (active M = 10, SD = 4; rest M = 12, SD = 4) (*F*_1,10_ = 0.59, *P* = 0.461) (Fig. 2C). We found strong evidence that uric acid was lower during the rest phase (M = 0.79, SD = 0.3) in comparison to the active (M = 1.39, SD = 0.5) (*F*_1,10_ = 13.00, *P* = 0.005) (Fig. 2D).

### Effects of sampling date, order, sex, time of sample, and handling time

The only evidence found for the effect of sampling date was found in SOD, which increased in both states as sampling date increased (*F*_1,20_ = 12.01, *P* = 0.002). We also found evidence that sampling order in relation to OXY (*F*_1,12_ = 11.75, *P* = 0.003) and the GSH:GSSG ratio (*F*_1,12_ = 5.67, *P* = 0.003), both of which were higher for the second sample in comparison to the first. We found evidence in the active samples that sex had an effect on oxidized glutathione (GSSG), which was higher in females (M = 232, SD = 29) than males (M = 200, SD = 15) (*F*_1,14_ = 6.02, *P* = 0.0278). No evidence was found for any effects of time of sample or handling time on any of the markers of oxidative stress. Please see Supplementary Material for more information.

## Discussion

Contrary to our expectation, ROS damage was found to be significantly lower during the period of heightened activity when compared to samples taken from birds out of rest. This is in direct contrast to the traditional thinking that increased energy metabolism will lead to an increase in ROS production and thus the accumulation of ROS damage. Daytime energy use in great tit females engaging in parental care is known to be several times higher than nighttime resting metabolism (Daan et al. 1990; Bryant and Tatner 1991; Tinbergen and Verhulst 2000; Zagkle 2022). Despite the higher metabolism during the Active phase than during the Rest, increased damage caused by ROS is not reflected in our comparative analysis. This adds to the growing body of evidence suggesting that increased metabolism does not necessarily cause a direct increase in ROS damage (Speakman et al. 2015) and adds strength to the claim that key foundations on which the oxidative stress model is based on are critically flawed (Speakman and Garratt 2014).

### Enzymatic antioxidants

One possible explanation for ROS damage to be higher in birds sampled out of rest compared to females sampled out of active food provisioning of their offspring was built into the second hypothesis we wished to examine, that birds have evolved antioxidant counter measures to compensate for increased ROS production during periods of heightened energy use. To test this hypothesis, we examined the levels of the three “first line” antioxidant enzymes, SOD, GPx, and CAT, along with the low weight antioxidant glutathione (GSH, GSSG, and GSH:GSSG ratios) but found no significant differences between the rest and active phases in any of the measurements. It has previously been shown in migrating European robins (*Erithacus rubecula*) that birds flying at night had higher levels of GPx activity than those caught during the day during stopover while foraging/resting (Jenni-Eiermann et al. 2014). The authors also found levels of oxidative damage to be highest during the nocturnal migration, which to the best of our knowledge is the only other study to measure free living birds during daytime and nighttime, albeit their energy usages are opposite to ours when time is considered. That study likely shows a species-specific upregulation of GPx in preparation for migration whereas birds in our study have presumably been performing at the peak of their capabilities for brood rearing and their enzyme activity levels results likely indicate the maximum levels obtained during reproduction. Zebra finches had increased GPx activity following 6 weeks of flight training as opposed to control birds (Cooper-Mullin et al. 2019) and also long-distance horse (*Equus ferus caballus*) training programs can increase antioxidant enzymes (particularly GPx) in red blood cells (Janiak et al. 2010). It is probable that our birds reached maximal levels of these first line defense enzymes early in the breeding season and maintain them throughout the summer, although we did find a relationship between higher SOD activity with increasing sample date, which may illustrate an increase toward the end of the breeding season. Indeed, it is likely that each enzyme may respond differently to activity over time and even exhibit its own circannual patterns of expression. Although the timeline in which the processes of ROS production, antioxidant upregulation and ROS neutralization occur is still unknown (McWilliams et al. 2021).

### Nonenzymatic antioxidant capacity

Similar to the antioxidant enzyme levels, our results for total circulating nonenzymatic antioxidant capacity, as measured by the OXY test, cannot explain our ROS damage results, our result of oxidative damage is not mirrored in the measures of the antioxidative capacity. Previous experimental results relating to nonenzymatic antioxidant capacity to workload have shown a decrease of 19% in homing pigeons (*Columba livia*) flown for 200 km in comparison to 60 km (Costantini et al. 2008). Comparisons between energy expenditure types such as long distance flying and food provisioning for young should be taken with caution, while both require exhaustive metabolic requirements, sources of energy are differing as one set of animals is powering food provisioning activities by regular daily food intake while many migrants use stored bodily resources (see below for the effect of metabolic state on oxidative status, as well as McWilliams et al. 2021).

One other aspect of the antioxidant defenses mechanisms in birds is the presence of uric acid, the activity of which is not included and thus measured in the OXY test (Costantini 2011). Levels of uric acid should be measured concurrently given that its origin and function differ from the of other antioxidants (Costantini et al. 2011a; Skrip and McWilliams 2016). Our results show levels of circulating uric acid were significantly higher during the active phases as opposed to the rest phase, a not entirely surprising result given the knowledge on uric acid production in relation to protein turnover outlines below. Uric acid acts as a powerful scavenger of singlet oxygen, peroxyl and hydroxyl radicals in the hydrophilic environment (Sautin and Johnson 2008); it has been demonstrated to inhibit lipid peroxidation (Smith and Lawing 1983) and may thus help explain the differences seen in oxidative damage.

Uric acid is the final product of protein catabolism in birds and the increased levels seen in circulation during the active phases may be seen in result of increased protein turnover during daytime (Clugston and Garlick 1982). Birds lack the enzyme urate oxidase, which oxidizes uric acid to allantoin; however, allantoin is present in avian plasma (Simoyi et al. 2003). Uric acid molecules, acting as nonenzymatic oxidants quench ROS and in doing so are converted to allantoin prior to excretion.

One source for the emergence of uric acid in the system is through dietary protein breakdown. White throated sparrows (*Zonotrichia albicollis*) fed insect diet (51% protein) had significantly higher levels of uric acid than those fed grain or fruit diets (10% protein each) (Smith et al. 2007), so it seems directly related to higher protein uptake, and our birds do certainly differ in uptake with zero uptake during the night and any food related uptake during daytime activity. Another source of uric acid is via catabolic breakdown of protein products in response to work, in our case potentially food provisioning, as a means to repair and replace worn out tissue. In white-crowned sparrows (*Zonotrichia leucophrys gambelii*), it has been demonstrated that post-exercise plasma levels of UA and allantoin are significantly correlated (Tsahar et al. 2006), leading to the authors to speculate that “one of the consequences of increased protein catabolism during prolonged exercise may be an improved antioxidant defense resulting from the higher UA concentration,” a prediction also speculated at in Klaassen et al. (2000) and a phenomenon likely occurring in our birds as well. This result would seem to prove our second hypothesis that birds have evolved antioxidant counter measures to compensate for increased ROS production during periods of heightened energy; however, this antioxidant capacity is not measured through the OXY assay but rather through confirmation of the protein breakdown.

### Effects of individuality, circadian/diurnal patterns, and metabolic states on oxidative status

In relation to ROM concentration, all but one of the individual birds in this study showed decreased levels from the rest to the active samples, while OXY values were far more variable. These individualistic responses can be based on several factors, which may be hard to control in the field, including time at last meal (and composition of meal itself), previous predator or specific encounters, and/or environmental effects such as pollutants or adverse weather conditions (Speakman et al. 2015). Under controlled conditions, repeat sampling in greenfinches (*Carduelis chloris*) has shown that several oxidative stress markers exhibit marked individual consistency. These included plasma carotenoids, total antioxidant capacity (TAC) and erythrocyte GSH, which were significantly correlated at the same time of day 8 days apart, while plasma OXY, oxidative damage (malondialdehyde), and uric acid were not, and none showed this relationship after 16 days (Sepp et al. 2012). The same authors note that data on individual consistency of biomarkers of oxidative stress have seldom been reported and highlights an area where future studies may focus.

For many behavioral and physiological traits, daily patterns are known to exist in birds, for example, melatonin levels, song production and locomotor behavior, and many of those are in fact circadian (Cassone 2014; Markowska et al. 2017). These traits have a pattern, which is not only concurrent but likely linked to feeding and fasting rhythms in association with overall energy metabolism, at is highly probable that the oxidative system is also part of this daily pattern (Hardeland et al. 2003).

In free living animals, however, researchers are yet to confirm whether time of day is an important confounding factor for evaluation of oxidative status (Skrip and McWilliams 2016), and variation seen in results may be a failure to take into account the time of the measurement (Blount et al. 2016). Few studies have been published which have examined daily rhythms in oxidative stress markers. One study found increasing antioxidant capacity (OXY) in nestling blue petrels (*Halobaena caerulea*) with increasing sample time (Costantini and Bonadonna 2010), whole in another higher GPx activity and oxidative damage was seen during nocturnal migration of European robins in relation to sample time before and after dawn, which might be related to past migration versus rest (Jenni-Eiermann et al. 2014). In jungle bush quail (*Perdicula asiatica*), a marked 24-h rhythm was found in both SOD and CAT activity, with both found to peak during the night, several hours before lights on, coinciding with the peak of melatonin. An inverse relationship was seen with oxidative damage (malondialdehyde), which peaked late in the day before falling in the middle of the night and rising again toward lights on. These rhythms were seen in both lung and thyroid tissues (Kharwar and Halder 2012, Verma et al. 2016, respectively), with differences in the timing of these peaks found when comparing birds in the reproductively active and inactive periods, likely due to adjusted photoperiods between seasons.

One possible confounding factor in relation to the oxidative status of the individuals within their daily rhythms are corticosterone (CORT) levels. Glucocorticoids (GC) may have a significant effect on oxidative stress, although this depends on duration of treatment with chronic stress having a larger impact than acute stressors; however, evidence in favor of the pro-oxidant effects of GCs is mixed (reviewed in Costantini et al. 2011b). Owing to the presence of GC receptors within mitochondria, it has been suggested that levels of GC may modulate mitochondrial metabolic activities, although this modulation is biphasic. Short acute exposure to stress hormones is associated with mitochondrial biogenesis and enzymatic activity of selected subunits of the ETC, whereas chronic stress may cause ETC dysfunction leading to increased ROS generation (Manoli et al. 2007). Rather than confounding effects on oxidative status markers during capture and sampling in the active phase, we believe that GC may explain the heightened levels of oxidative damage seen during the rest phase. GCs are known to exhibit a distinct unimodal circadian rhythm with more corticosterone released at the end of the dark phase with a trough being reached throughout the day, as seen in Gambel’s white crowned sparrows (*Zonotrichia leucophrys gambelii*) (Breuner et al. 1999) and great tits (Carere et al. 2003).

The hypothalamic–pituitary–adrenal (HPA) axis is responsible for the release of CORT in response to handling and restraint (Li et al. 2019). This HPA axis is most sensitive just after light on and as such we believe the compounding effects of CORT on oxidative status will be most pronounced during the night when levels are naturally high rather than on samples taken during activity when levels are low and the HPA response is weak (Breuner et al. 1999). Elevated GC levels are associated with protein turnover and breakdown, although tissue protein maybe protected at baseline GC levels associated with the physiological state B (predictable seasonal levels for a given life-history stage) as opposed to effects seen in state C (emergency life-history stage) and high levels just prior to the active phase likely promote locomotor activity and resumption of feeding (Landys et al. 2006). While much is known relating to daily patterns of hormones in animals, such as melatonin or CORT as described, this same predictive is lacking for oxidative stress markers. Indeed, it is highly likely that these same hormones are playing a role in the oxidative status of animals and it maybe that our sampling times (∼3 and ∼10 AM) and the results seen are inextricably linked to hormonal levels at or before these times and shifting these sampling times a few hours either way could possibly alter the results found in our measures of oxidative status. For example, the clear peaks of SOD and CAT seen in the jungle quail described above (Kharwar and Halder 2012, Verma et al. 2016), occurring several hours before light-on but not seen in our ∼3 to 10 AM comparison.

Birds are especially relevant to studies such as these as they have incredibly segregated active and rest periods compared to mammals. With the exception of nocturnal/crepuscular birds (and those undertaking nocturnal migration), avian biological clocks are markedly associated with activities such as feeding and sleeping (switching to rest phase almost immediately after lights out and are then post absorptive after roughly an hour), whereas mammals typically do not exhibit such compulsory day/night rhythms and often have staggered patterns of behavior including food consumption (Breuner et al. 1999). Differences between mammals and birds in these regards maybe attributable to their different biological master clocks (Bell-Pederson et al. 2005). The mammalian circadian timing system is orchestrated by the suprachiasmatic nucleus (SCN) of the hypothalamus, part of the HPA axis described above, the rhythmicity of which is present in constant darkness, but which be entrained to light (Hasting et al. 2019). In birds, this pacemaker system is more complex, with the pineal gland as the master pacemaker, responsible for melatonin production which prompts rest. Pineal activity is strongly linked to light/dark cues, which it receives through both the retinal via the SCN and direct extraocular stimulation via the skull by at least four distinct brain structures, which are functionally photoreceptive (Cassone 2014; Markowska et al. 2017).

Despite the low energy metabolism during rest compared to active periods, it is reasonable to assume that a considerable amount of ROS is still being generated as the bird’s metabolism is idling while resting at night. This ROS production, coupled with low levels of the circulating antioxidant activity of uric acid may explain the results of ROS damage we have seen. Our birds are consuming caterpillars as their main diet during the summer months, and it is likely that the low levels of uric acid seen during rest are reflective of baseline post-absorptive levels in comparison to those seen during activity while the birds are actively catabolising protein from dietary insects and performing intra-cellular protein turnover.

Our sampling schedule was designed to determine the oxidative state of birds at diametric and extreme periods of energetic metabolism, while at rest following a full night of recuperation and when being most active during food provisioning. We attempted to discern the patterns of as many different aspects of the oxidative systems of our birds under the limitations of repeat sampling and amount of blood, which could be collected. The results of our study were opposite to our working hypothesis that oxidative damage will be higher during activity than during rest. These results point to gaps in our understanding of the effects of energy use on the oxidative status of animals. Some of these gaps maybe due to differences in treatments, tissues, and tests, which are used by various researchers; however, another gap in our understanding of this subject maybe due simply to the lack of data on the effects of sampling time, that is, failure to take into account the daily activity of feeding rhythm or hormonal activities and the likely impacts this has on results. We believe this is an area, which may provide exciting opportunities for discovery in future studies.

## Conclusions

Based on our surprising results of higher oxidative stress levels in females sampled out of rest compared to birds sampled out of active food provisioning several conclusions can be forwarded:

1. The link between energy use and oxidative stress is not as straightforward as expected. Higher energy use may, in theory, relate to higher ROS production but not necessarily result in increased oxidative damage, as seen in our direct Rest versus Active analysis of oxidative damage levels. This agrees with the statement that current models of oxidative stress generation and trade-offs may be too simplistic and not fully engulfing the complexity of oxidative stress with its multitude of evolved prevention defense mechanisms (Speakman and Garratt 2014). It is likely that several compounding factors (time of sample, metabolic state, etc.) are in action concurrently and should be taken into account in a more wholistic approach.
2. While much is known about the patterns of hormones in animals associated with the day/night cycle, the same cannot be said for patterns of oxidative stress markers. Patterns in the redox system likely relate to hormonal fluctuations, patterns, and cycles, which is even less investigated and thus understood. Birds are an excellent system for investigations on such links as these as they exhibit an incredible strong segregation between active and rest phases. Potential future studies could aim to vary time of sampling and include this in analysis to gain a better knowledge of the daily modulations of oxidative stress.
3. The metabolic state of the animal, that is, post-absorptive or feeding and/or anabolic or catabolic is relevant to its oxidative status at time of sampling, as such should be included in any analysis. Metabolic state likely determines, or strongly influences, the intrinsic temporal organization of an organism. Thus, we believe that time of sampling within 24 h may prove to be an important predictor for oxidative status.
4. Uric acid appears to be playing a vital role in the oxidative status of birds. Acting as a potent antioxidant within birds, heightened levels of uric acid produced during the waking hours appear to negate any potential increase in ROS production protecting the birds from the accumulation of ROS damage. This conclusion is in line with other studies and our repeated sampling schedule and the associated expectable differences in uric acid may provide new rational of why uric acid—as the potent antioxidant that it is to be considered—would be able to have such a strong impact. The levels of uric acid may also be linked to the temporal aspect of sampling within the day–night cycle, but this temporal aspect of sampling may also account for other antioxidants or prooxidants (however, point 3 must also be seen independently).
5. Last but not least, our study provides further evidence that the interpretation of the oxidative stress index (d-ROMs/OXY × 1000) must be taken with care, particularly if the result is negative, like in the present study. Potentially, the role of uric acid as an antioxidant not accounted for by the OXY-adsorbent assay (Costantini 2011) may also play an explanatory role here.

## Supplementary Material

icad120_Supplemental_File

## Data Availability

Data and R scripts used for analysis are freely available from the Jagiellonian University Repository available through RODBUK at https://uj.rodbuk.pl/dataset.xhtml?persistentId=doi%3A10.57903%2FUJ%2FJ8CRIH&version=DRAFT.
