# Supplementary material for: Repeat Sampling of Female Passerines During Reproduction Reveals Surprising Higher Plasma Oxidative Damage During Resting Compared to Active State
Source: Integr Comp Biol. 2023 Sep 12;63(6):1197–208. doi: 10.1093/icb/icad120 (PMC10755187; doi:10.1093/icb/icad120)
Supplement: icad120_Supplemental_File [file icad120_supplemental_file.docx]

**Electronic Supplementary Materials**

**Materials, methods and results**

**Main title:**

**Repeat sampling of female passerines during reproduction reveals surprising higher plasma oxidative damage during resting compared to active state**

**Running Title:**

**Repeat sampling of passerines**

Kyle Coughlan^1*^, Edyta T. Sadowska^1^, Ulf Bauchinger^1,2^

^1^ Institute of Environmental Sciences, Jagiellonian University, Gronostajowa 7, 30-387 Kraków, Poland

*^2^ Nencki Institute of Experimental Biology*, *Polish Academy of Sciences*, 3 Pasteura St., Warsaw, Poland

*****Corresponding author: kyle.coughlan@doctoral.uj.edu.pl

K. Coughlan (https://orcid.org/0000-0002-0760-7214)

E.T. Sadowska (https://orcid.org/0000-0003-1240-4814)

U. Bauchinger (https://orcid.org/0000-0002-6422-3815)

Materials and methods

*Study area, nest boxes and species*

In 2019 nest boxes were installed in the forests within and surrounding the Nencki Institute Research Station close to the town of Mikołajki situated in the Masurian Lake Distract in Northern Poland (N 53° 47′ 09.86″ E 21° 34′ 53.26″). The nest boxes measure (135x135x350 mm) with a 3 cm entrance hole. Nest boxes were attached to tree with the entrance hole roughly 1.5 m from the ground and have a removable roof section for ease of checking and access. There are 500 nest boxes laid out in a 50x50 m grid covering 137 ha. The enclosed confines of the station contain 76 nest boxes within an area of roughly 17 ha. The forest within the station is assessed as fresh mixed forest rich with species. The five most represented species constituting 82.15% of the stand are pine (*Pinus sylvestris,* 44.21%, up to 180 years old), maple (*Acer platanoides,* 11.25%), liden (*Tilia cordata*, 9.78%), spruce (*Picea abies*, 9.58%) and oak (*Quercus robur,* 7.33%), and the rest of the stands are made up of 26 other species of trees and shrubs. The remaining nest boxes are spread out amongst 120 ha of forests surrounding the station. These forests are less diverse, mainly consisting of pine plantations for agroforestry interspersed with various patches of deciduous trees but also contains areas of swampy ground, large open clearings (natural and clearcuts), and fields. The study area is bordered to the north by the town of Mikołajki, the east by the lake of Mikołajki (Jezioro Mikołajskie), the west by a road and the south by farmland and other forest patches.

This study investigated the breeding biology and oxidative status of the passerine bird, the great tit (*Parus major,* Linnaeus, 1758). Great tits are obligatory secondary cavity breeders building nests in natural tree cavities and those previously excavated by woodpeckers, but they readily breed in nest boxes when provided for them. They are facultative secondary round breeders and often produce a second clutch within the same summer. This study relates to the second round of reproduction for 15 great tit nests from early June to early August 2021 and measures the antioxidant levels for birds during two different levels of physical activity, i.e. at Rest and while Active.

*Field methods*

Nest boxes were monitored throughout the 2021 breeding season starting in early April. Nest boxes were checked weekly for presence of nest material. Upon confirmation of a nest these boxes were checked daily to confirm start of laying date (date of first laid egg), egg number and hatching date (hatching day = Day 1 for the nest).

Upon confirmation of hatching each nest was assigned to sampling times on Day 10 and Day 12. Nest were sampled at night and morning on Day 10 and Day 12 alternatively, i.e., the first nest was assigned a night sampling on Day 10 and morning sampling on Day 12, the second nest morning sampling Day 10 and night sampling Day 12, and so on with the rest of the nests. On the occasion an animal was unable to be caught on its assigned day this was then attempted on the next, full details provided below in “*Number and details of samples”* section.

*Catching procedures*

For the Resting samples the nest was approached before the start of civil twilight and females removed from the nest using red light head torches. Sampling was attempted to be undertaken as close to half an hour before civil twilight as possible, actual average sampling time in relation to civil twilight for the morning of sampling was -25 mins ± 19 mins, while real time average sample time was 03:06 AM ± 12 mins. The nests were approached quietly using head lamps with red light. The lids were opened, and female were taken directly from the nest. Upon catching birds were moved a distance away from the nest and their blood was sampled (See *Blood sampling* below for details). Once this was completed, they were replaced in their nest with their chicks.

For the morning sample birds were caught in their nests using a “foil trap”, this is a clear piece of thin but rigid plastic which is placed inside the nest and held in place using a thumbtack in the top corners. This was positioned to cover roughly half of the entrance hole with a finger wide gap on the bottom to allow the birds to see, smell and hear the chicks encouraging them to enter (Fig. 1). It requires a little force from the birds to push the plastic foil up allowing for their entrance at which point the foil returns to position behind them sealing them in.

These traps were placed in order to sample as close to civil twilight +7 hours as possible, actual average sampling time in relation to civil twilight for the day samples were taken on was civil twilight + 6 h 46 mins ± 23 mins., while real time average sample time was 10:17AM ± 18 mins. Once the traps were in place the observers left the vicinity of the nest box and an observer returned ever 15 to 20 mins to check if the birds had entered the nest and the trap was left in place a maximum of one hour. Upon catching birds were moved a distance away from the nest and their blood was sampled (See *Blood sampling* below for details). If the male was caught before the female, he was sampled while the trap remained in place, whereas if the female was caught first the trap was removed immediately, while on one occasion both sexes were present in the nest during the same check. After sampling birds were released on site and observers left the area.

Each time a bird was removed from their nest at night, or upon removal of the foil trap during morning sampling the number and condition of the chicks was recorded.

*Measurements and ringing*

Once birds were in the hand either during night or morning sampling they were placed in a cloth bag and taken a short distance away from their nests. Birds which had not been previously ringed were given metal ID rings on their left legs while females had large yellow plastic rings with ID numbers added to their right legs. During the first sampling morphometric measurements were taken including tarsus and wing length, while birds were aged in the day light during the morning sampling according to plumage and notes were taken on extent of moulting if occurring. Body mass was measured each time a bird was in hand, using an electronic scale accurate to 0.1g. On Day 14 of the nest chicks were ringed and had their body mass, wing and tarsus length measured.

*Blood sampling and storage*

Samples were collected following measurements of the bird by piercing the brachial vein and collecting blood in heparinized 75 µl heparinized capillary tubes. Two tubes were collected, and blood was blood was transferred to a heparinized 0.5 ml Eppendorf tube which was stored on ice for transport back to the research station where the blood was centrifuged (g x 6,000 for 10 mins) to separate erythrocytes from plasma. The erythrocytes and plasma sample were separated and stored on dry ice in a polystyrene box inside a freezer at -20°C until the end of the field season when they were transported on dry ice to the Jagellonian University in Krakow and stored at -80 °C until analysis.

*Number and details of samples*

In total 12 females were sampled at both times points; one female was not present in her nest for night sampling of Day 12 and so was sampled at night of Day 13; one female was not present for night sampling on Day 10 and so was sampled Day 11 and subsequently the morning sampling was done on Day 13, while another was not caught during the morning of Day 10 but was caught on Day 11 with the following night sample taken on Day 13; one female was sampled in the night on Day 10 but was not captured with trap on morning of Day 12 or Day 13 and so is represented by a single measurement. In addition, seven males were captured during the foil trapping during the morning sample collection. Further explanation of samples and statistical modelling is proved below in the “*Statistical Analysis”* section*.*

*Laboratory analysis*

*Erythrocyte antioxidant enzymatic and non-enzymatic activity*

Activity levels for three enzymes were analysed using erythrocytes, these were Glutathione Peroxidase (GPx), Superoxide dismutase (SOD), and Catalase (CAT). These three enzymes constitute the first line of antioxidant defence (Ighodaro and Akinloye 2018). The main area of activity for these enzymes is within and around the mitochondria where the majority of reactive oxygen species (ROS) are produced, mostly through aerobic respiration and ATP production across the inner mitochondrial membrane, for this reason erythrocytes were used to quantify the levels on enzyme activity. Additionally, glutathione levels were measured from the erythrocyte lysate. Glutathione is a non-enzymatic tripeptide and the most common thiol present in animal cells.

Following the separation of erythrocytes and plasma as described above, the erythrocytes were subjected to a protocol for quantification of the mentioned enzymes and glutathione. All values were quantified using commercially available kits from Cayman Chemicals (Ann Arbor, USA) in which erythrocytes required the same initial processing. In brief, the erythrocyte pellet was lysed with addition of 4x ice-cold distilled H_2_O and centrifuging at g x 10,000 for 15 minutes at 4 °C. This action lyses the cells releasing the contents and the resulting supernatant was removed and used for the assays, this supernatant is here after referred to as the sample for the erythrocyte assay kits.

*Superoxide Dismutase*

Superoxide Dismutase (SOD) are a group of metallonenzymes whose main function is to catalyse the dismutation of the superoxide radical (O_2_^-^) into molecular oxygen (O^2^) and hydrogen peroxide (H^2^O^2^). The reaction catalysed by SOD is extremely fast and the presence of sufficient amounts of the enzyme in cells and tissues typically keeps the concentration of the superoxide radical very low.

Erythrocyte enzymatic activity levels of SOD were measured using the Superoxide Dismutase Assay kit (Item No. 706002, Cayman Chemicals, Ann Arbor, USA) following the manufacturers protocol. First, 0.5 μl of sample was diluted 1:200 with sample buffer (50 mM Tris-HCL, pH. 8.0). 10 μl of this diluted sample was added to each well along with 200 μl of diluted Radical Detector (made of 50 μl tetrazolium salt solution diluted with 19.95 ml of assay buffer; 50 mM Tris-HCL, pH. 8.0 containing 0.01 mM diethylenetriaminepentaacetic acid (DTPA) and 0.1 mM hypoxanthine). The reaction was initiated with addition of 20 μl of diluted Xanthine Oxidase to the wells. The plate is then incubated at 25 °C for 30 minutes with mild shaking (75 rpm). Following incubation absorbance values for the plate were read in a spectrophotometer at 450 nm (Tecan infinite m200 with associated Tecan i-control 2.0 software).

Calibration of SOD activity was calculated using a titration of known SOD Standard (bovine erythrocyte SOD (Cu/Zn)). The calibration curve of 7 points with a blank as the final point and each point was run in duplicate per plate. Absorbance values for each point (minus the blank absorbance) for each duplicate were averaged to produce the final linearized rate (LR) of SOD activity by dividing the standard point A absorbance by itself and those of the other standards (i.e LR for Std A = Abs Std A/Abs Std A; LR for Std B = Abs Std A/Abs Std B; LR for Std C = Abs Std A/Abs Std C etc.). The equation for this LR were then used for sample SOD activity values.

Final SOD activity of the sample is expressed as of unit of SOD activity per ml of sample; U/ml. One unit is defined as the amount of enzyme needed to exhibit 50% dismutation of the superoxide radical. These values were calculated based on the above described LR using the following equation:

$$SOD \left( U/ml \right)=\left[ ( \frac{Sample LR\pm\left( y-intercept \right)}{Slope} )x \frac{0.23ml}{0.01ml} \right] x sample dilution$$

Samples were run in duplicate on the same plate and the average of two duplicates was used for analysis. Intraplate coefficients of variation were 5.1%.

*Glutathione peroxidase*

Glutathione peroxidase (GPx) is an enzyme which catalyses the reduction of hydroperoxides (including hydrogen peroxide, H_2_O_2_) and protects cells from oxidative damage. It utilizes reduced glutathione as an electron donor to regenerate selenocysteine within the enzyme structure which acts as a powerful agent and reduces lipid hydroperoxides to alcohols and free hydrogen peroxide to water.

Erythrocyte enzymatic activity levels of GPx were measured using the Glutathione Peroxidase Assay kit (Item No. 703102, Cayman Chemicals, Ann Arbor, USA) following the manufacturers protocol. First, 5 μl of sample was diluted 1:15 with sample buffer (50mM Tris-HCL, ph. 7.6 containing 5mM EDTA and 1 mg/ml BSA). 20 μl of this diluted sample were added per well along with 50 μl of assay buffer (50 mM Tris-HCL, pH. 7.6, containing 5 mM EDTA), 50 μl of Co-Substrate mixture (containing lyophilized glutathione and glutathione reductase (GR) and 50 μl NADPH. The reaction was initiated by addition of 20 μl Cumene Hydroperoxide and the plate was incubated 25 °C for 1 minutes with mild shaking (75 rpm). A coupled reaction occurs with GPx and GR whereby oxides glutathione (GSSG) is produced via the reduction of hydroperoxides by GR and is recycled to its reduced state (GSH) by GR and NADPH. The oxidation of NADPH to NADP^+^ is accompanied by a decrease of light absorbance when measured at 340 nm. This decrease is directly proportional to GPx activity in the sample. Calibration of GPx activity was calculated using the absorbance of a blank of distilled H_2_O while a positive control of glutathione peroxidase (bovine erythrocyte GPx) was used to confirm correct functioning of the assay. Absorbance values for the plate were read in a spectrophotometer at 340 nm (same model as described above). Values were measured every 30 seconds for 5 minutes and enzymatic activity was calculated based on the decrease of absorbance (given in ΔA_340_). Erythrocyte GPx activity values are given as nmol of GPx per minutes and ml of sample; nmol/min/ml; and were calculated using the following equation.

$$GPx activity =\frac{\Delta A_{340}/min}{0.0037 \mu M^{-1}} x\frac{0.19ml}{0.02ml} x Sample diltuion$$

Samples were run in duplicate on the same plate and the average of two duplicates was used for analysis. Intra-coefficients of variation were 2.1%.

*Catalase*

Catalase (CAT) is a ubiquitous antioxidant enzyme present in nearly all aerobic cells and is involved in the detoxification of hydrogen peroxide (H_2_O_2_), breaking it down into water and oxygen. The CAT enzyme catalyses the conversion of two H_2_O_2_ molecules to molecular oxygen and two molecules of water. It also exhibits a peroxidatic activity in which low molecular weight alcohols can serve as electron donors. While CAT utilizes aliphatic alcohols as specific substrates other enzymes with peroxidatic activity do not.

Erythrocyte enzymatic activity levels of CAT were measured using the Catalase Assay kit (Item No. 707002, Cayman Chemicals, Ann Arbor, USA) following the manufacturers protocol. First, 0.5 μl of sample was diluted (1:400) with assay buffer (100 mM potassium phosphate, pH 7.0). then, 20 μl of this dilute sample was added per well along with 30 μl methanol. The reaction was initiated by added 20 μl H_2_O_2_ to all wells and the plate was incubated at 25 °C for 20 minutes with mild shaking (75 rpm). Then 30 μl Potassium Hydroxide was added to terminate the reaction. 30 μl Catalase Purpald as chromogen (4-amino-3-hydrazino-5mercapto-1,2,4-triazole in 0.5 M hydrochloric acid) is added and the plate undergoes another incubation at 20 °C for 10 minutes with mild shaking (75 rpm). Finally, 10 μl of Catalase Potassium Periodate was added and the plate was incubated 25 °C for 5 minutes with mild shaking (75 rpm). Following incubation absorbance values for the plate were read in a spectrophotometer at 540 nm (same model as above).

The CAT enzyme reacts with methanol in the presence of an optimal concentration of H_2_O_2_ and produces formaldehyde which is measured colourimetrically via the chromogen which forms a bicyclic heterocycle with aldehydes which upon oxidation changes from a colourless substance to a purple coloured one, the intensity of which is directly related to catalase activity. Calibration of CAT activity was calculated using a titration of known formaldehyde concentration to produce a standard curve and a positive control of Catalase (bovine liver CAT). The calibration curve of 7 points with a blank as the final point and each point was run in duplicate per plate. Absorbance values for each point (minus the blank absorbance) for each duplicate were averaged to produce the final calibration curve equation with sample absorbance (minus the blank) calculated using the following equation:

$$Formaldehyde \left( \mu M \right)=\left[ \frac{Sample abs\pm\left( y-intercept \right)}{Slope} \right]x\frac{0.17ml}{0.02ml}$$

Final CAT activity of the sample is expressed as nmol of CAT activity per minutes and ml of sample; nmol/min/ml, and were calculated using the following equation:

$$CAT activity=\left[ \frac{\mu M of Sample}{20 minutes} \right] x Sample dilution$$

Samples were run in duplicate on the same plate and the average of two duplicates was used for analysis. Intraplate coefficients of variation were 4.7%.

*Glutathione measurements*

Glutathione is a non-enzymatic antioxidant and acts by neutralising (i.e., reducing) reactive oxygen species and free radicals. Glutathione exists in two states in the cells, reduced glutathione (GSH) and oxidised glutathione disulfide (GSSG). The conversion of GSH to GSSG occurs in the presence of ROS and free radicals and is catalysed by the enzyme glutathione reductase. The ratio of GSH to GSSG is used as a common biomarker of cellular oxidative stress. First samples were measured for total glutathione tGSH, and then inhibition of reduced glutathione for exclusive measurement of GSSG. Reduced glutathione;GSH was calculated as tGSH minus GSSG

First, the samples were subject to deproteination. It is necessary to remove as much protein as possible from the samples to avoid interferences due to particulates and sulfhydryl groups on proteins in the assay. This was done by taking 2 μl of lysate and adding an equal volume of MPA reagent (5g of metaphosphoric acid (Sigma Aldrich Item no 239275) mixed with 50ml of H_2_O) to each sample and vortexing. After 5 minutes standing at room temperature this mixture was centrifuged at g x 2,000 for 3 minutes and the supernatant was collected. Next TEAM reagent was added (50 μl per ml of sample) made up of a 4 M solution of triethanolamine (Sigma Aldrich Item no T58300) which was 531 μl of triethanolamine mixed with 469 μl of H_2_O.

50 μl of sample and 50 μl of standard were added to their respective wells followed by addition of 150 μl of reaction cocktail (MES buffer 11.25 ml, reconstituted Cofactor mixture 0.45 ml, reconstituted Enzyme mixture 2.1 ml, water 2.3 ml and reconstitute DTNB 0.45ml). The plate was incubated at 25 °C for 25 minutes with mild shaking (75 rpm). Following incubation absorbance values for the plate were read in a spectrophotometer at 410 nm (same model as above).

Oxidised glutathione (GSSG) was calculated using the same protocol as described above expect for processing of the samples prior to assay to inhibit the reduced glutathione in the samples. This was done by derivatizing GSH in the samples with a 1M solution 2-vinylpyridine (Sigma Aldrich Item no 13229-2) allowing for the exclusive measurement of GSSG following which the samples were assayed.

Samples were run in duplicate on the same plate and the average of two duplicates was used for analysis. Intraplate coefficients of variation were 0.4% for tGSH and 0.8% for GSSG.

*Plasma oxidative status*

*Plasma reactive oxygen metabolites (ROMs)*

Reactive oxygen species (ROS) are highly reactive with macromolecules such as lipids, proteins and nucleic acid which can maintain oxidising properties such as reactive oxygen metabolites (ROMs). These chemicals (e.g., hydroperoxides, ROOH) are capable of propagating the oxidation cascade and the increase of ROM concentration within the circulating plasma system is a consequence of the release of pro-oxidants from intracellular compartment or from an inflammatory process via and immune response. The presence of metal such as iron (Fe^2+^ and Fe^3+^) and copper (Cu^+^ and Cu^2+^) promote the toxicity of ROM by catalysing the cleavage of ROOH resulting in the generation of two pro-oxidants, namely the alkoxyl (R-O•) and alkylperoxyl (R-OO•), both of which are highly reactive and histolesive.

Plasma concentration of ROMs was measured using the d-ROMs kit (Diacron International, Grosseto Italy) with a modified protocol (Costantini and Dell-Omo 2006). First, 4 μl of plasma was added to a well and diluted with a 200 μl “working solution” made of 100 parts 0.1 M acetic acid/sodium acetate buffer (pH. 4.8) to 1 part chromogen. The plate was incubated at 37 °C for 75 minutes with mild shaking (75 rpm). Similar to the OXY test ROMs an alkyl-substituted aromatic amine solubilised in the chromogen which produces a pink colour, however, opposed to the OXY test the colour intensity produced by the reaction in the d-ROMs test is directly proportional to the concentration of ROM contained in the plasma. Following incubation, the plate is read in the spectrophotometer at 505 nm (same model as above). Using a titration of a known calibration serum a standard curve was used to calculate the concentration of ROMs in the plasma samples by inserting the absorbance of the samples into the standard curve equation. Results are expressed in an arbitrary unit called “Carratelli Units” (UCARR). The calibration curve of 7 points ranged from 4.375 to 140 UCARR with a blank as the final point and each point was run in duplicate per plate and was based on a provided standard of known concentration. Absorbance values for each point (minus the blank absorbance) for each duplicate averaged to produce the final calibration curve equation, and sample UCARR concentration were calculated based on sample absorbance (minus the blank) using the following equation:

$$UCARR=\frac{Sample Abs. \pm(y-intercept)}{Slope}$$

The values expressed in UCARR were converted based on the following rational, 1 UCARR is equivalent to the oxidizing potential of 0.08 mg hydrogen peroxide H_2_O_2_ per dl. These UCARR values were converted using the factor of 0.08 mg H_2_O_2_ dl = 0.02352 mM/l H_2_O_2_ with final values given in mM/l H_2_O_2_ equivalent concentration of ROMs in the plasma. Samples were run in duplicate on separate plates using 4 μl dilution for each replicate and the average of the two duplicates was used for analysis. Inter-plate coefficients of variation were 7.8%.

*Plasma antioxidant capacity*

Defence against harmful ROS and free radicals is mediated by the plasma antioxidant barrier. This barrier contains “sacrificial” elements which are non-active chemicals with antioxidant properties present in the plasma. This barrier includes both exogenous (e.g. ascorbate, tocopherols and carotenoids) and endogenous (e.g uric acid) chemicals. Plasma antioxidant capacity was measured using the OXY Adsorbent kit (Diacron International, Grosseto Italy) using a modified protocol (Costantini and Dell'Omo 2006). This kit uses colorimetric determination based on the potential of the plasma antioxidant barrier to inhibit hypochlorous acid (HCIO). This is a chemical has massive oxidant action potential and is a pro-oxidant of pathologic relevance in biological systems. First, 2 μl of plasma sample was diluted with 200 μl of distilled H_2_O and vortexed for 10 seconds. 2 μl of this diluted plasma is added to a well with 200 μl of HCIO based oxidant solution and incubated at 37 °C for 10 minutes with mild shaking (75 rpm). After incubation 2 μl of *N*,*N*-diethyl-*p*-phenylenediamine as chromogen is added to each well. The HCIO remaining in the sample oxidises an alkyl-substituted aromatic amine solubilised in the chromogen whose transformed derivative possesses a pink colour. The intensity of this colour is inversely related to the antioxidant capacity of the plasma when measured in a spectrometer at 490 nm (Tecan infinite m200 with associated Tecan i-control 2.0 software). Calibration of reading was achieved using a blank of 2 μl distilled H_2_O and 2 μl diluted plasma serum (2 μl serum of known concentration in 200 μl distilled H_2_O) in duplicate per plate with the average values used for sample antioxidant capacity calculation using the following formula:

$$\frac{(Abs regagent blank-Abs sample)}{(Abs reagent blank-Abs calibrator)} x [Calibrator]$$

Measurements are expressed as μmol/ml of HCIO neutralised. Samples were run in duplicate on separate plates using a fresh 2 μl plasma to 200 μl distilled H_2_O dilution for each replicate and the average of the two replicates was used for analysis. Inter-plate coefficients of variation were 6.4%.

*Plasma uric acid concentration*

Uric acid is waste product produced via nitrogen metabolism which can act as an antioxidant by inhibiting lipid peroxidation and protecting DNA from radical damage. It has been demonstrated previously that uric acid contained within the plasma does not correlate significantly with the plasma antioxidant barrier as measured by the OXY Adsorbent test described above. As such independent results of the OXY test and uric acid concentration in the plasma can be evaluated together to produce a more rounded pictures of the total antioxidant defences of the plasma barrier (Costantini 2011).

Plasma uric acid concentration was measured using the Uric Acid kit (Diacron International, Grosseto Italy) with a modified protocol for smaller plasma volumes (in essence the manufacturer’s protocol with all reagents and samples ÷ 4). First, 2.5 μl of plasma sample is added per well and diluted with 100 μl of buffer solution. This solution is made by mixing 2 reagents, the first is a buffer solution of TRIS buffer 60.0 mmol/l and 2,4,diclorophenol-sulphonate (DHBS) 2.0 mmol/l which is used to dissolve the second reagent made up of Uricase ≥ 090 U/l, peroxidase ≥ 1000 U/l and 4-aminophenazone 0.8 mmol/l with no reactive stabilizers. The plate is then incubated at 37 °C for 5 minutes with mild shaking (75 rpm). During incubation uric acid is converted to allantoin and hygrogen peroxide by uricase. Under the catalytic influence of peroxidase, the hydrogen peroxide reacts with 4-aminophenazone and DHBS which forms a red coloured compound. Following incubation, the plate is read in the spectrophotometer at 510 nm (same model as above), at this wavelength the intensity of the red colour compound is directly proportion to the concentration of uric acid in the sample. Calibration of reading was achieved using a blank of 2.5 μl distilled H_2_O and 2.5 μl uric acid standard of known concentration in duplicate per plate with the average values used for sample antioxidant capacity calculation using the following formula:

$$Uric acid \left[ \frac{mg}{dl} \right]=\frac{EC}{ESTD}x Conc STD.$$

Where EC is the absorbance of the sample – absorbance of the blank. ESTD is the absorbance of the calibrator- absorbance of the blank. Values are given in mg/dl of uric acid concentration of the plasma samples. Samples were run in duplicate on separate plates using 2.5 μl plasma for each replicate and the average of the two duplicates was used for analysis. Inter-plate coefficients of variation were 5.6%.

All assay plates were measured using a Tecan infinite m200 plate reader with associated i.control 2.0 software.

*Statistical analysis*

Statistical analyses were performed using RStudio ver. 3.6.2. A mixed model approach was taken using the lme4/lmerTest packages in RStudio (Bates et al. 2015). We tested several different models based on the data collected. Oxidative stress index was calculated as d-ROMs/OXY x 1000, according to Costantini et al. 2008. The GSH:GSSG ratio calculated according to Owen and Butterfield 2010.

1. *Repeated female differences between activity and rest phase (same as main manuscript)*

All ten measures of oxidative status were used as individual response variables with State (i.e. Rest or Active) used as the explanatory variable. Sampling date and order of sample were included as covariates in repeated measures along with ring number as a random factor.

1. *Rest phase separate analysis*

A linear model approach was taken with each of the ten measurements of oxidative status. Sampling date and time of sample were included as covariates.

1. *Active phase separate analysis*

All ten measures of oxidative status were used as individual response variables with sampling date, sex, time of sample and handling time included as covariates. Nest box number was included as a random factor to account for couples effects.

*Results*

Results for the difference in females between the active and rest phase are presented in the main manuscript, while Fig. 2. and Fig. 3. below presents them graphically. The mean values and standard deviations for all measurements taken both during Rest at night and during Activity during the day are presented below in Table 1. All statistical results for the above-described models have been laid on in Table 2 below.

**References:** Please note the references listed here are numbered correspond to the above table and not the main manuscript.

1. Ighodaro OM, Akinloye OA. 2018. First line defence antioxidants-superoxide dismutase (SOD), catalase (CAT) and glutathione peroxidase (GPX): Their fundamental role in the entire antioxidant defence grid. Alexandria J Med. 54(4), 287-293. (doi: 10.1016/j.ajme.2017.09.001)
2. Costantini D, Dell'Omo G. 2006. Environmental and genetic components of oxidative stress in wild kestrel nestlings (*Falco tinnunculus*). J Comp Physiol B. 176(6):575-9. (doi: 10.1007/s00360-006-0080-0)
3. Costantini D. 2011. On the measurement of circulating antioxidant capacity and the nightmare of uric acid. Methods Ecol Evol. 2(3), 321-325. (doi: 10.1111/j.2041-210X.2010.00080.x)
4. Bates D, Mächler M, Bolker B, Walker S. 2015. Fitting Linear Mixed-Effects Models using lme4. *J. Stat. Softw*. 67(1), 1–48. (doi: 10.18637/jss.v067.i01)
5. Costantini D, Dell'ariccia G, Lipp HP. 2008. Long flights and age affect oxidative status of homing pigeons (*Columba livia*). J Exp Biol. 211(Pt 3):377-81. (doi: 10.1242/jeb.012856)
6. Owen JB, Butterfield DA. 2010 Measurement of oxidized/reduced glutathione ratio. Methods Mol Biol. 648:269-77. (doi: 10.1007/978-1-60761-756-3_18)


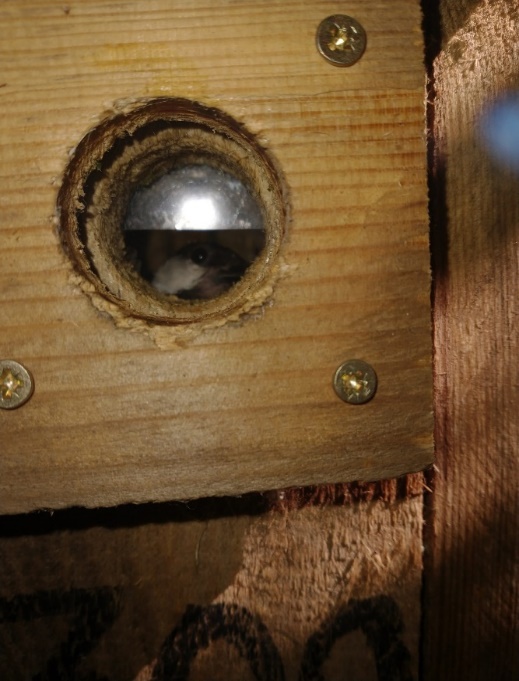

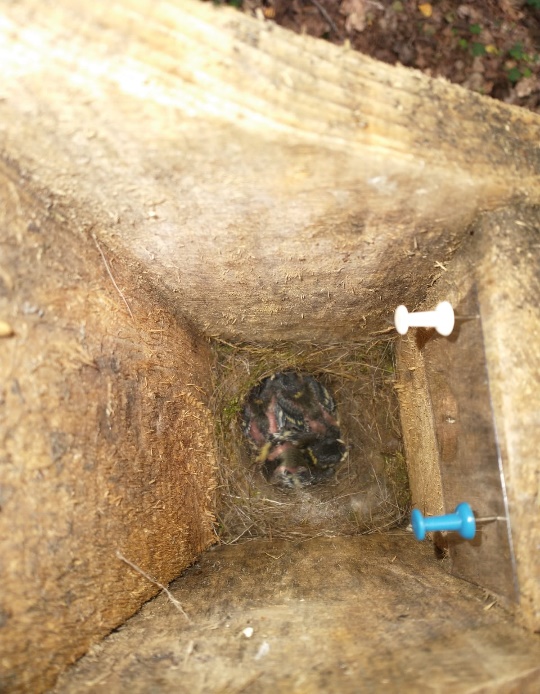


**Fig. 1**. Foil traps. The “foil” is a thin piece of rigid plastic held in place using two thumbtacks at the top corners inside the nest. As can be seen the plastic extends halfway down the entrance hole allowing the birds to see, smell and hear their chicks clearly. They can enter via going down and under pushing the foil up which then closes behind them. Left: Male great tit captured using the foil trap at NB309. Right: Foil trap in nest before lid placement.


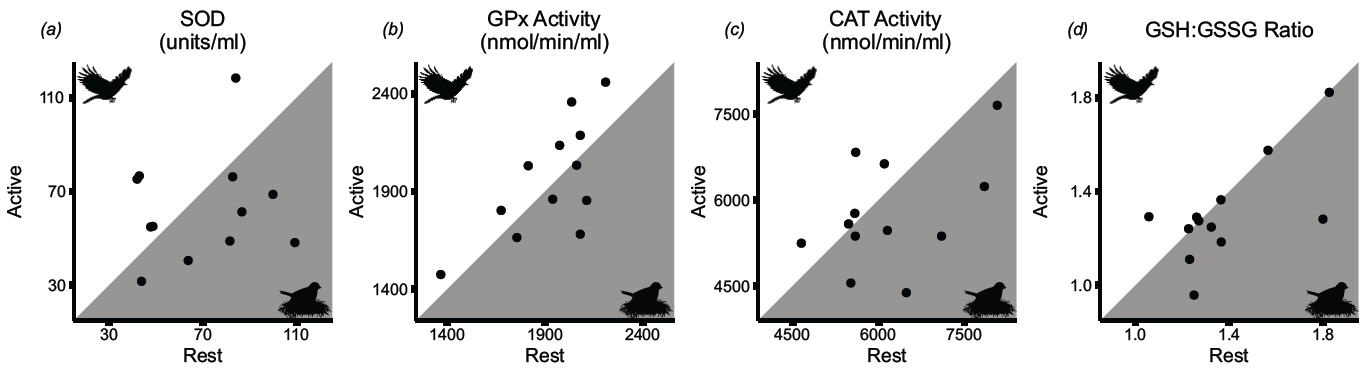


**Fig. 2.** Antioxidant activity of erythrocyte lysate. Flying birds in top left of each panel represent the active phase values while bird sitting in nest in bottom right represent the rest phase. Samples which are higher during active phase appear above the diagonal while those higher during rest appear below. The three “first line” defence antioxidants (a) SOD (sodium dismutase), (b) GPx (glutathione peroxidase) and (c) CAT (catalase) showed no significant differences between the active and rest phases. Glutathione in its reduced (GSH) and oxidised form (GSSG) also did not exhibit any differences between rest and active phases and is exhibited in the ratios between GSH and GSSG (d).


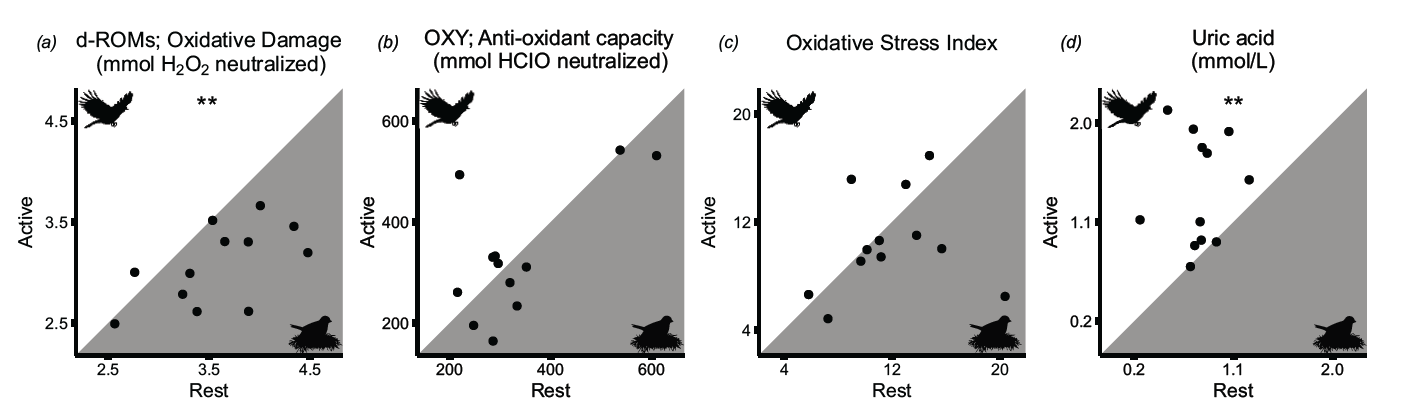


**Fig. 3.** Plasma oxidative stress markers. Flying birds in top left of each panel represent the active phase values while bird sitting in nest in bottom right represent the rest phase. Samples which are higher during active phase appear above the diagonal while those higher during rest appear below. *(a)* d-ROMs, our measure of oxidative damage was significantly higher during the night rest phase than during the daytime activity, with all but one sample higher during the day (*p* = 0.006); *(b)* OXY, non-enzymatic antioxidant capacity of plasma, showed no significant effect of activity state, as did *(c)* OSI, oxidative stress index (d-ROMs/OXY*1000). *(d)* Uric acid, a potent antioxidant present in plasma, showed a significant effect of activity state being far higher in during daytime activity than during the rest phase (*p* = 0.005).

**Table 1:** Mean and standard deviation values for all measurements of oxidative stress taken during the Rest phase (night) and Active phase (day) in repeated female samples.

|  | Measurement | Rest phase value  (Mean ± SD) | Active phase value  (Mean ± SD) |
| --- | --- | --- | --- |
| d-ROM’s | mmol H2O2 neutralized | 3.59 ± 0.6 | 3.08 ± 0.4 |
| OXY | mmol HCIO neutralized | 332.59 ± 120.8 | 332.76 ± 126.1 |
| OSI | d-ROM’s/OXY*1000 | 11.81 ± 4.0 | 10.41 ± 3.7 |
| Uric Acid | mmol/L | 0.79 ± 0.3 | 1.39 ± 0.5 |
| SOD | units/ml | 69.38 ± 24.1 | 63.00 ± 22.6 |
| GPx | activity; nmol/min/ml | 1925.35 ± 235.9 | 1961.99 ± 293.1 |
| CAT | activity; nmol/min/ml | 6181.06 ± 1027.6 | 5758.07 ± 942.6 |
| GSH | µM | 315.93 ± 32.4 | 300.72 ± 54.1 |
| GSSG | µM | 233.12 ± 32.5 | 232.08 ± 29.3 |
| GSH:GSSG ratio | GSH/GSSG | 1.38 ± 0.2 | 1.30 ± 0.2 |
|  | | | |

|  | Female Repeat Samples  (n=12 birds*2) | | | Rest Samples  (n=13) | | | Active Samples  (n=19 (12F/7M)) | | |
| --- | --- | --- | --- | --- | --- | --- | --- | --- | --- |
|  |  | | |  | | |  | | |
|  |  | | |  | | |  | | |
|  | d.f. | *F-* | *p*-value | d.f. | *F* | *p*-value | d.f. | *F* | *p*-value |
| dROM’s |  |  |  |  |  |  |  |  |  |
| State | 9.99 | 12.12 | **0.0059**** |  |  |  |  |  |  |
| Sampling Date | 10.04 | 0.95 | 0.3532 | 1 | 1.62 | 0.2321 | 12.95 | 0.04 | 0.8434 |
| Order | 11.16 | 0.32 | 0.5840 |  |  |  |  |  |  |
| Sex |  |  |  |  |  |  | 9.17 | 2.11 | 0.1794 |
| Time of Sample |  |  |  | 1 | 3.53 | 0.0898 | 13.09 | 1.79 | 0.2037 |
| Handling Time |  |  |  |  |  |  | 12.01 | 1.79 | 0.2053 |
| OXY |  |  |  |  |  |  |  |  |  |
| State | 10.00 | 0.11 | 0.7518 |  |  |  |  |  |  |
| Sampling Date | 10.07 | 3.32 | 0.0985 | 1 | 0.33 | 0.5763 | 5.96 | 0.46 | 0.5242 |
| Order | 11.75 | 6.17 | **0.0291*** |  |  |  |  |  |  |
| Sex |  |  |  |  |  |  | 8.81 | 0.13 | 0.9103 |
| Time of Sample |  |  |  | 1 | 0.43 | 0.5284 | 6.92 | 0.47 | 0.5171 |
| Handling Time |  |  |  |  |  |  | 6.69 | 0.09 | 0.7680 |
| OSI |  |  |  |  |  |  |  |  |  |
| State | 10.00 | 0.59 | 0.4608 |  |  |  |  |  |  |
| Sampling Date | 10.03 | 0.11 | 0.7464 | 1 | 0.66 | 0.4364 | 6.45 | 0.96 | 0.3632 |
| Order | 10.66 | 3.23 | 0.1004 |  |  |  |  |  |  |
| Sex |  |  |  |  |  |  | 9.19 | 0.00 | 0.9512 |
| Time of Sample |  |  |  | 1 | 2.15 | 0.1730 | 6.97 | 1.50 | 0.2603 |
| Handling Time |  |  |  |  |  |  | 7.82 | 0.70 | 0.4273 |
| Uric Acid |  |  |  |  |  |  |  |  |  |
| State | 10.00 | 13.00 | **0.0048**** |  |  |  |  |  |  |
| Sampling Date | 10.01 | 0.01 | 0.9246 | 1 | 0.83 | 0.3849 | 14 | 0.47 | 0.5061 |
| Order | 10.43 | 0.64 | 0.4410 |  |  |  |  |  |  |
| Sex |  |  |  |  |  |  | 14 | 0.02 | 0.8776 |
| Time of Sample |  |  |  | 1 | 0.03 | 0.8601 | 14 | 0.10 | 0.7608 |
| Handling Time |  |  |  |  |  |  | 14 | 0.23 | 0.6389 |

**Table 3:** Results for plasma oxidative status markers for all statistical models run in the analysis, significant results are presented in **bold.**

**Table 3:** Results for erythrocyte antioxidant enzymes for all statistical models run in the analysis, significant results are presented in **bold.**

|  | Female Repeat Samples  (n=12 birds*2) | | |  | | | Active Samples  (n=19 (12F/7M)) | | |
| --- | --- | --- | --- | --- | --- | --- | --- | --- | --- |
|  | d.f. | *F* | *p*-value | d.f. | *F* | *p*-value | d.f. | *F* | *p*-value |
| SOD |  |  |  |  |  |  |  |  |  |
| State | 20.00 | 0.46 | 0.5062 |  |  |  |  |  |  |
| Sampling Date | 20.00 | 12.01 | **0.0024*** | 1 | 14.48 | **0.0035**** | 14 | 0.81 | 0.3846 |
| Order | 20.00 | 1.61 | 0.2197 |  |  |  |  |  |  |
| Sex |  |  |  |  |  |  | 14 | 0.01 | 0.9371 |
| Time of Sample |  |  |  | 1 | 3.28 | 0.1001 | 14 | 0.43 | 0.5220 |
| Handling Time |  |  |  |  |  |  | 14 | 0.12 | 0.7319 |
| GPX |  |  |  |  |  |  |  |  |  |
| State | 10.00 | 0.42 | 0.5311 |  |  |  |  |  |  |
| Sampling Date | 10.07 | 0.51 | 0.4920 | 1 | 0.01 | 0.9355 | 9.70 | 3.98 | 0.0750 |
| Order | 11.76 | 0.07 | 0.7982 |  |  |  |  |  |  |
| Sex |  |  |  |  |  |  | 7.79 | 0.20 | 0.6674 |
| Time of Sample |  |  |  | 1 | 0.00 | 0.9646 | 9.88 | 0.93 | 0.3586 |
| Handling Time |  |  |  |  |  |  | 9.86 | 0.38 | 0.8500 |
| CAT |  |  |  |  |  |  |  |  |  |
| State | 9.99 | 1.48 | 0.2522 |  |  |  |  |  |  |
| Sampling Date | 10.02 | 1.87 | 0.2008 | 1 | 2.09 | 0.1787 | 10.8 | 0.52 | 0.4864 |
| Order | 10.83 | 0.52 | 0.4858 |  |  |  |  |  |  |
| Sex |  |  |  |  |  |  | 9.998 | 1.28 | 0.2837 |
| Time of Sample |  |  |  | 1 | 0.10 | 0.7552 | 10.97 | 0.00 | 0.9549 |
| Handling Time |  |  |  |  |  |  | 11.3 | 1.37 | 0.2656 |
| GSH |  |  |  |  |  |  |  |  |  |
| State | 9.98 | 2.40 | 0.1524 |  |  |  |  |  |  |
| Sampling Date | 10.03 | 0.07 | 0.7937 | 1 | 0.72 | 0.4167 | 13.3 | 0.36 | 0.5563 |
| Order | 11.20 | 3.82 | 0.0761 |  |  |  |  |  |  |
| Sex |  |  |  |  |  |  | 10.6 | 0.37 | 0.5577 |
| Time of Sample |  |  |  | 1 | 3.397 | 0.0744 | 13.4 | 1.70 | 0.2149 |
| Handling Time |  |  |  |  |  |  | 12.7 | 2.24 | 0.1587 |
| GSSG |  |  |  |  |  |  |  |  |  |
| State | 9.98 | 0.03 | 0.8708 |  |  |  |  |  |  |
| Sampling Date | 10.09 | 2.50 | 0.1448 | 1 | 2.36 | 0.1543 | 14 | 3.67 | 0.7614 |
| Order | 12.14 | 0.27 | 0.6111 |  |  |  |  |  |  |
| Sex |  |  |  |  |  |  | 14 | 6.02 | **0.0279*** |
| Time of Sample |  |  |  | 1 | 0.13 | 0.7276 | 14 | 1.00 | 0.3338 |
| Handling Time |  |  |  |  |  |  | 14 | 1.27 | 0.2786 |
| GSH:GSSG ratio |  |  |  |  |  |  |  |  |  |
| State | 9.99 | 3.43 | 0.0939 |  |  |  |  |  |  |
| Sampling Date | 10.06 | 1.85 | 0.2035 | 1 | 0.37 | 0.5562 | 12.8 | 2.00 | 0.1814 |
| Order | 11.91 | 5.67 | 0.0349 |  |  |  |  |  |  |
| Sex |  |  |  |  |  |  | 11.0 | 1.93 | 0.1918 |
| Time of Sample |  |  |  | 1 | 1.07 | 0.3264 | 12.9 | 5.34 | **0.0380*** |
| Handling Time |  |  |  |  |  |  | 12.5 | 0.55 | 0.4723 |
